# Supplementary material for: ProteinShader: illustrative rendering of macromolecules
Source: BMC Struct Biol. 2009 Mar 30;9:19. doi: 10.1186/1472-6807-9-19 (PMC2672931; doi:10.1186/1472-6807-9-19)
Supplement: Additional file 1 — ProteinShader program without source code. This compressed file contains the complete ProteinShader program including associated libraries, but no source code. A README.txt file gives an overview of the ProteinShader distribution, and the index.html file in the help subdirectory has directions on getting started with the program as well as a set of tutorials. [file 1472-6807-9-19-S1.zip › ProteinShader-beta-0_9_4-binary/help/api/org/proteinshader/gui/listeners/controlpanel/ColorPanelListenerFactory.html]

ColorPanelListenerFactory (ProteinShader API)


|  |  |  |
| --- | --- | --- |
| **Overview** \| **Package** \| **Class** \| **Use** \| **Tree** \| **Deprecated** \| **Index** \| **Help** | |  |
| **PREV CLASS**   **NEXT CLASS** | **FRAMES**    **NO FRAMES**     **All Classes** |  |
| SUMMARY: NESTED \| FIELD \| CONSTR \| METHOD | DETAIL: FIELD \| CONSTR \| METHOD |  |


---


## org.proteinshader.gui.listeners.controlpanel Class ColorPanelListenerFactory

```
java.lang.Object
  org.proteinshader.gui.listeners.controlpanel.ColorPanelListenerFactory
```

---

``` public class ColorPanelListenerFactory extends Object ```

Creates the listener for the ColorPanel.

---

**Constructor Summary**

`ColorPanelListenerFactory(Mediator mediator,
ColorPanel colorPanel)`
  
          Constructs a ColorPanelListenerFactory.


| **Method Summary** | |
| --- | --- |
| `ActionListener` | `createChooserButtonActionListener()`             Creates an action listener for the "Chooser" button. |
| `ChangeListener` | `createChooserChangeListener()`             Creates a ChangeListener that will get the color selection from the JColorChooser and calls on the ColorPanel to change the color of whatever items are currently selected. |
| `ActionListener` | `createDefault1ButtonActionListener()`             Creates an action listener for the first default button. |
| `ActionListener` | `createDefault2ButtonActionListener()`             Creates an action listener for the second default button. |

**Methods inherited from class java.lang.Object**

`clone, equals, finalize, getClass, hashCode, notify, notifyAll, toString, wait, wait, wait`

**Constructor Detail**

### ColorPanelListenerFactory

```
public ColorPanelListenerFactory(Mediator mediator,
                                 ColorPanel colorPanel)
```

:   Constructs a ColorPanelListenerFactory.

    **Parameters:**: `mediator` - the centralized Mediator object that listeners call on to accomplish their task.: `colorPanel` - the ColorPanel that this class creates listeners for.


**Method Detail**

### createDefault1ButtonActionListener

```
public ActionListener createDefault1ButtonActionListener()
```

:   Creates an action listener for the first default button. When the
    action listener is called, it will apply the default 1 color to
    whatever objects are currently selected in the RadioPanel.

    :   **Returns:**: The action listener.

---


### createDefault2ButtonActionListener

```
public ActionListener createDefault2ButtonActionListener()
```

:   Creates an action listener for the second default button. When
    the action listener is called, it will apply the default 2 color
    to whatever objects are currently selected in the RadioPanel.

    :   **Returns:**: The action listener.

---


### createChooserButtonActionListener

```
public ActionListener createChooserButtonActionListener()
```

:   Creates an action listener for the "Chooser" button. When the
    action listener is called, it will open a JColorChooser. Any
    color selections made by the JColorChooser will be used to enter
    numbers in the ColorPanel text fields for Red, Green, and Blue.

    :   **Returns:**: The action listener.

---


### createChooserChangeListener

```
public ChangeListener createChooserChangeListener()
```

:   Creates a ChangeListener that will get the color selection from
    the JColorChooser and calls on the ColorPanel to change the color
    of whatever items are currently selected.

    :   **Returns:**: The action listener.


---


|  |  |  |
| --- | --- | --- |
| **Overview** \| **Package** \| **Class** \| **Use** \| **Tree** \| **Deprecated** \| **Index** \| **Help** | |  |
| **PREV CLASS**   **NEXT CLASS** | **FRAMES**    **NO FRAMES**     **All Classes** |  |
| SUMMARY: NESTED \| FIELD \| CONSTR \| METHOD | DETAIL: FIELD \| CONSTR \| METHOD |  |


---

# *Copyright © 2007-2008*
